# The Long-Term Benefit of Sacubitril/Valsartan in Patients with HFrEF: A 5-Year Follow-Up Study in a Real World Population

**DOI:** 10.3390/jcm12196247

**Published:** 2023-09-28

**Authors:** Giuseppe Dattilo, Giulia Laterra, Roberto Licordari, Francesca Parisi, Lorenzo Pistelli, Luigi Colarusso, Luca Zappia, Vittoria Vaccaro, Elisabetta Demurtas, Marta Allegra, Pasquale Crea, Gianluca Di Bella, Salvatore Santo Signorelli, Nadia Aspromonte, Egidio Imbalzano, Michele Correale

**Affiliations:** 1 Section of Cardiology, Department of Biomedical and Dental Sciences and Morphofunctional Imaging, University of Messina, 98122 Messina, Italy; giuseppe.dattilo@unime.it (G.D.); robertolicordari@gmail.com (R.L.); 2 Department of Clinical and Experimental Medicine, Policlinic University Hospital of Messina, 98122 Messina, Italy; giulilaterr@unime.it (G.L.); francesca6973@gmail.com (F.P.); pis.lorenzo@gmail.com (L.P.); luigcolarus@polime.it (L.C.); luczappi@unime.it (L.Z.); vittorivaccar@polime.it (V.V.); elisabetdemurta@polime.it (E.D.); martallegr@unime.it (M.A.); pasqualecrea85@gmail.com (P.C.); gianluca.dibella@unime.it (G.D.B.); 3 Internal Medicine Unit, Department of Clinical and Experimental Medicine, University of Catania, 95125 Catania, Italy; santo.signorelli@unict.it; 4 Department of Cardiovascular Medicine, Fondazione Policlinico Universitario A. Gemelli IRCCS, 00168 Rome, Italy; nadia.aspromonte@policlinicogemelli.it; 5 Cardiothoracic Department, Policlinico Riuniti University Hospital, 71100 Foggia, Italy; michele.correale@libero.it

**Keywords:** Sacubitril/Valsartan (LCZ696), heart failure, heart failure with reduced ejection fraction (HFrEF), chronic heart failure, real-world study

## Abstract

Heart failure (HF) is a progressive condition with an increasing prevalence, and the scientific evidence of heart failure with reduced ejection fraction (HFrEF) reports a 6% rate of 1-year mortality in stable patients, whereas, in recently hospitalized patients, the 1-year mortality rates exceed 20%. The Sacubitril/Valsartan (S/V), the first angiotensin receptor neprilysin inhibitor (ARNI), significantly reduced both HF hospitalization and cardiovascular mortality. Aim of the study: to evaluate the effect of S/V in a follow-up period of 5 years from the beginning of the therapy. We compared the one-year outcomes of S/V use with those obtained after 5 years of therapy, monitoring the long-term effects in a real-world population with HFrEF. Methods: Seventy consecutive patients with HFrEF and eligible for ARNI, according to PARADIGM-HF criteria, were enrolled. All patients had an overall follow-up of 60 months, during which time they underwent standard transthoracic echocardiography (TTE) with Global Longitudinal Strain (GLS) evaluation, the Kansas City Cardiomyopathy Questionnaire (KCCQ), the Six Minutes Walking Test (6MWT), and blood tests (NT-pro-BNP and BNP, renal function tests). Results: NTproBNP values were reduced significantly among the three time-points (*p* < 0.001). Among echocardiographic parameters, left ventricle end-diastolic volume (LV EDV) and E/e’ significantly were reduced at the first evaluation (12 months), while left ventricle end-systolic volume (LV ESV) decreased during all follow-ups (*p* < 0.001). LV EF (*p* < 0.001) and GLS (*p* < 0.001) significantly increased at both evaluations. The 6MWT (*p* < 0.001) and KCCQ scores (*p* < 0.001) increased significantly in the first 12 months and remained stable along the other time-points. NYHA class showed an increase in class 1 subjects and a decrease in class 3 subjects during follow-up. NTproBNP, BNP, 6MWT, and KCCQ scores showed a significant change in the first 12 months, while LVEF, GLS, and ESV changed during all evaluations. Conclusions: We verified that the improvements obtained after one year of therapy had not reached a plateau phase but continued to improve and were statistically significant at 5 years. Although our data should be confirmed in larger and multicentre studies, we can state that the utilization of Sacubitril/Valsartan has catalysed substantial transformations in the prognostic landscape of chronic HFrEF, yielding profound clinical implications.

## 1. Introduction

Heart Failure (HF) has a prevalence in the world of approximately 1–2% in the adult population [1]. The prevalence of HF is increasing (by 23% over the past decade) mainly due to the ageing of the population, with the age-specific incidence [2,3,4,5].

In the ESC HF guidelines, HF is classified into three distinct phenotypes based on the measurement of left ventricular ejection fraction (LVEF) (FE < 40% HFrEF, FE 41–49 HFmrEF, FE > 50% HEpEF). Clinical trials of HFrEF report a 6% rate of 1-year mortality, whereas, in large, registry-based surveys, 1-year mortality rates exceed 20% in patients recently hospitalized for HF but are closer to 6% in those recruited with stable outpatient HF [6]. Furthermore, patients with HF who show impaired functional capacities experience a declined ability to carry out their daily activities and suffer a reduced quality of life [7,8].

In the last few years, the prognosis and the quality of life in patients with HF has improved compared to the past thanks to new treatments.

The PARADIGM-HF trial showed that S/V (LCZ696) significantly reduced both HF hospitalization and cardiovascular mortality in comparison to guideline recommended doses of Enalapril [9]. These benefits are not modified by HFrEF aetiology [10]. In the recent ESC HF Guidelines [11], S/V is recommended as a replacement for an ACE-I in patients with HFrEF to reduce the risk of HF hospitalization and death.

We have recently demonstrated, in real-life data and in agreement with data from international literature, that the use of S/V in patients with HFrEF, and in maximal therapy according to guidelines, leads to an improvement in the perceived quality of life, increased resistance to physical effort, and an improvement in cardiac performance [12,13,14,15,16,17,18,19]. These improvements do not occur simultaneously over time, are independent of the aetiology of HF, are independent of the dose as long as the patient takes the maximum tolerated dose, and they are the same even in patients at high arrhythmic risk and taking high doses of antiarrhythmics and ICD carriers [12,13,14,15,16,17,18,19].

The aim of this study was to evaluate the effect of S/V in a follow-up period of 5 years from the beginning of the therapy. We compared the one-year outcomes of sacubitril/valsartan use with those obtained after 5 years of therapy, monitoring the long-term effects of sacubitril/valsartan in a real-world population with HFrEF previously treated with optimized therapy.

## 2. Materials and Methods

### 2.1. Study Population

Seventy consecutive patients with heart failure and reduced ejection fraction (HFrEF) and eligible for ARNI, according to PARADIGM-HF criteria, were enrolled in this prospective non-randomized observational study. The patients enrolled had an LVEF ≤ 35%, New York Heart Association (NYHA) class II–III symptoms, systolic blood pressure ≥ 100 mmHg, eGFR ≥ 30 mL/min/1.73 m^2^, and potassium levels ≤ 5.4 mmol/L. All enrolled patients were previously under treatment with the maximum tolerated dosage of beta-blockers, ACE inhibitors, and MRAs prior to commencing S/V at the time of enrolment. At the point of enrolment, the intake of ACE inhibitors was discontinued to initiate the administration of S/V. All patients were treated with S/V therapy in accordance with previous ESC HF Guidelines [11,20]. Before starting treatment with ARNI, all patients underwent a cardiological examination, and a 12-lead electrocardiogram was performed. All patients had an overall follow-up of 60 months, during which time they underwent (at baseline, after 12, and after 60 months of therapy) a standard transthoracic echocardiographic examination with Global Longitudinal Strain Measurements (GLS). They completed the Kansas City Cardiomyopathy Questionnaire (KCCQ) and performed the Six Minutes Walking Test (6MWT) and blood tests (NT-pro-BNP and BNP, renal function tests). The ARNI was started with a dose compatible with the patient’s clinical history and co-morbidities, according to specific prescription criteria. Then S/V treatment was established at the maximum tolerated dose (starting from 24/26 mg up to 97/103 mg twice a day). All patients gave their written informed consent, according to the Declaration of Helsinki.

### 2.2. Kansas City Cardiomyopathy Questionnaire (KCCQ)

The KCCQ is a self-administered questionnaire that quantifies multiple domains of patients’ HF-related health statuses, including physical limitations, symptom stability, symptom frequency, symptom burden, self-efficacy, quality of life, and social limitations. At the baseline and during follow-up, patients completed the form autonomously, guaranteeing, in this way, authentic answers [21,22]. An overall summary score can be derived. Values range from 0 to 100 with higher scores indicating better health status. A mean difference over time of 5 points on the KCCQ Summary score reflects a clinically significant change in heart failure status [23,24,25].

### 2.3. Echocardiography

Colour Doppler ultrasound examination was performed in each participant. The latest generation of ultrasound systems (GE E95, Horten, Norway) with phased-array 2.5–4.5 MHz transducers were utilized for the imaging. Two experienced cardiologists, who were blinded to patient clinical characteristics and examination timing, reviewed all digitalized echocardiographic images. All reported echocardiography measures were averaged from three consecutive cycles (or five if atrial fibrillation was present) in accordance with the recommendations of the American Society of Echocardiography and the European Association of Cardiovascular Imaging [26]. Baseline and follow-up echocardiography were performed on all patients using the same machine and acquisition protocol. End-diastolic volume (EDV) and end-systolic volume (ESV) were measured from the 4 + 2 chamber apical view, while left ventricular ejection fraction (LVEF) was calculated using the Simpson biplane method. The mitral flow velocities were recorded using an apical 4-chamber image, and pulsed wave Doppler sample velocities were placed and recorded for their ratio (E/A). Tissue Doppler imaging (TDI) was also performed to evaluate alternative left ventricular systolic and diastolic function parameters, such as S peak velocity (cm/s) and E/e’ ratio, with results expressed as mean values of septal and lateral measures.

To obtain the “off-line” measurement of Global Longitudinal Strain (GLS), a specific software suite (Echopac BT 12, GE, Horten, Norway) was used. The 17-segment model was employed to divide the LV (6 basal, 6 mid-level, 4 apical and the apical cap). Standard apical 4-, 3-, and 2-chamber views were used to automatically track the endocardial border of the LV on a tele-diastolic frame, which was manually adjusted when required. End-diastole and end-systole were defined by both ECG and visual assessment of 2D images, and the mean of three beats was taken for all measurements. The global strain was calculated using the mean value of 17 segments.

### 2.4. Six Minutes Walking Test

Six minutes walking test (6MWT) is a simple exercise test to evaluate physical functional capacity and clinical improvements in response to therapies, and it is widely used in HF patients [27,28]. We used a wide straight corridor that was 30 m in length. During this test, oxygen saturation, heart rate, and blood pressure were monitored at the beginning and at the end of the test. Patients were asked to walk as fast as possible with no restriction in resting for a while, if necessary. The 6MWT distance was calculated at baseline and during every follow-up.

### 2.5. Statistical Analysis

To examine whether variables had a normal distribution, the Kolmogorov–Smirnov test was utilized. Variables having a normal distribution were shown as mean ± SD, while non-normally distributed variables were shown as median and interquartile range. Repeated measures ANOVA with a Greenhouse–Geisser correction was used to compare clinical, echocardiographic, and laboratory features at baseline with the respective values at the 12 and 60 months follow-up visits. Post-hoc testing between the 3 time-points was performed using the Bonferroni correction for multiple comparisons. The same approach was used to study the evolution of the same features during follow-up based on aetiology, whether ischemic or idiopathic cardiomyopathy. A *p* < 0.05 was considered statistically significant. All statistical analysis was performed using RStudio (version 2022.07.2; Integrated Development Environment for R. Boston, MA, USA).

## 3. Results

The final population included 70 patients (60 males, 86%) with a mean age of 67 ± 10.6 years. The general characteristics of the population at baseline are shown in Table 1. In 66% of patients, the HF aetiology was ischemic, while, in 34%, it was addressed to an idiopathic dilated cardiomyopathy. Electric therapy was already started in 54 (77%) patients; in particular, 10 patients had a CRT, while 44 had an ICD.

### Follow-Up

**Figure 1:**
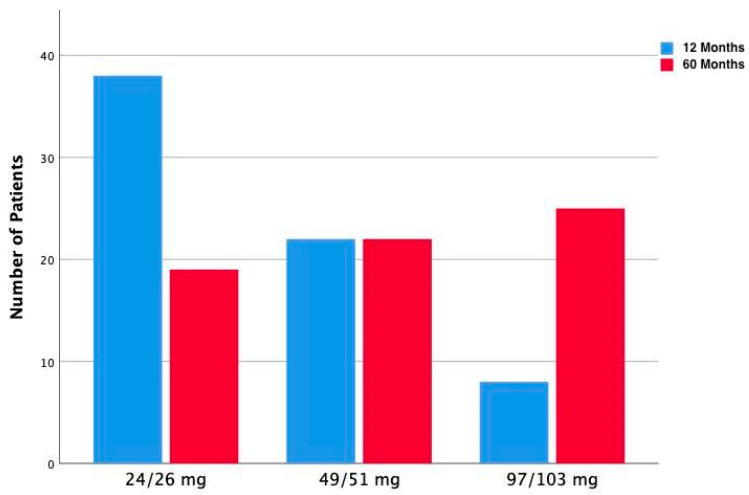
Bar graph depicting alterations in sacubitril/valsartan dosage prescriptions within the population at 12- and 60-month evaluations.

During a clinical follow-up at 60 months, one patient had serious cardiac events (one cardiac death, at 27 months from enrolment). At baseline, all patients were naïve to use of an angiotensin receptor neprilysin inhibitor; during the follow-up, all patients underwent sacubitril/valsartan prescription and up-titration as shown in Figure 1. In particular, at 60 months, 25 patients (38%) were treated with sacubitril/valsartan 97/103 mg, while, at the 12-month evaluation, only 8 patients (12%) were taking such a dosage.

Table 2 reports the characteristics of the patients and their changes during the three evaluations. SBP and DBP significantly declined in the first 12 months, while a mild rise in values was seen until the 60-month evaluation (*p* < 0.001). NTproBNP values decreased significantly among the three time-points (*p* < 0.001), while BNP values increased in the first 12 months (*p* = 0.008). Regarding echocardiographic parameters, LV EDV and E/e’ were significantly reduced at the first evaluation (12 months), while LV ESV decreased during all follow-ups (*p* < 0.001). LVEF (*p* < 0.001) and GLS (*p* < 0.001) significantly increased at both evaluations. The 6MWT (*p* < 0.001) and KCCQ scores (*p* < 0.001) increased significantly in the first 12 months and remained stable along the other time-points. NYHA class showed an increase in class 1 subjects and a decrease in class 3 subjects during follow-up.

**Figure 2:**
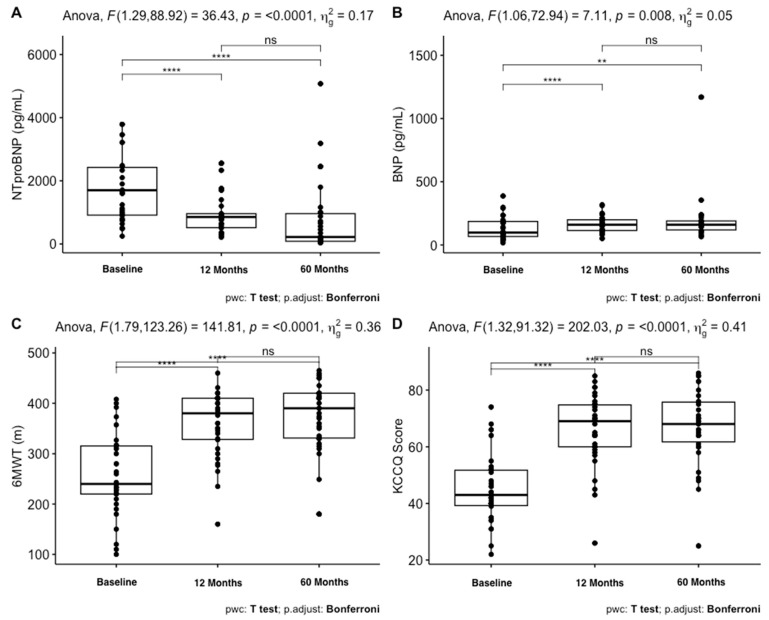
Box plots illustrating changes in various parameters throughout the follow-up period. (**A**) displays NTproBNP values, (**B**) shows BNP values, (**C**) presents Six Minutes Walking Test scores, and (**D**) exhibits KCCQ scores. *****: *p* < 0.0001, **: *p* < 0.001, ns: not significant.

**Figure 3:**
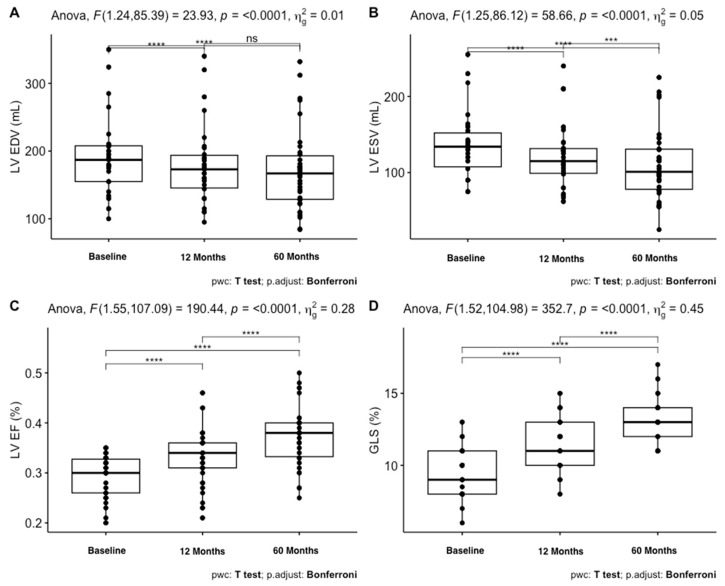
**Box plots highlighting variations in different parameters over the follow-up period.** (**A**) represents LV EDV values, (**B**) depicts LV ESV values, (**C**) shows LV EF, and (**D**) displays GLS values. *****: *p*: <0.0001, ***: *p*: <0.001, ns: not significant.

These results are better reported in Figure 2 and Figure 3. As shown in Figure 2, NTproBNP, BNP, 6MWT, and KCCQ scores had a significant change in the first 12 months, while LVEF, GLS, and ESV (Figure 3) changed during all evaluations.

**Figure 4:**
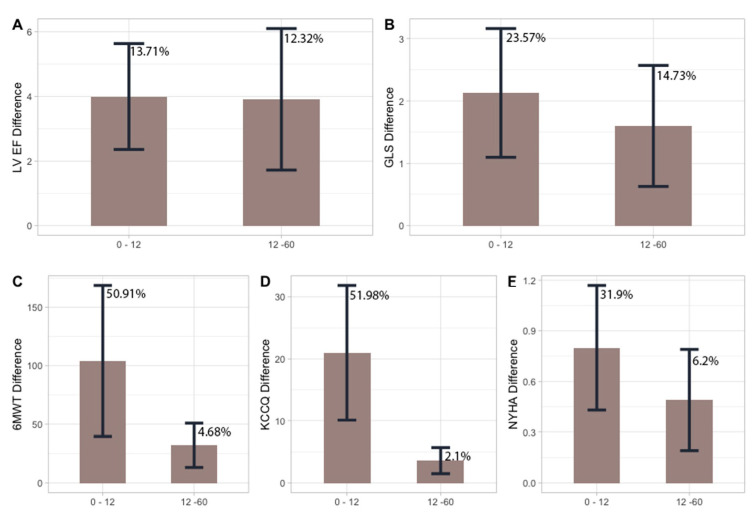
**Bar graphs indicating fluctuations during follow-up (baseline—12 months, 12 months–60 months).** Each bar represents the absolute variation in the respective parameter, with the percentage variation specified on each bar. Panel (**A**) illustrates LV EF variation during follow-up, Panel (**B**) shows GLS variation, Panel (**C**) displays 6MWT changes, Panel (**D**) presents KCCQ score alterations, and Panel (**E**) demonstrates NYHA class variations.

Table 3 reports differences in the same above-mentioned variables during follow-up, stratified by aetiology groups (ischaemic vs. idiopathic). DBP, NTproBNP, LV EF, GLS, 6MWT, KCCQ scores and NYHA class showed significant changes during follow-up in both groups. Fluctuations during follow-up (baseline—12 months, 12 months–60 months) are better reported in Figure 4. Each bar represents the absolute variation in the respective parameter, with the percentage variation specified on each bar.

## 4. Discussion

HF is a progressive condition with an increasing prevalence over the past decade. Scientific evidence of HFrEF reports a 6% rate of 1-year mortality in stable patients, whereas, in recently hospitalized patients, the 1-year mortality rates exceed 20% [2,3,4,5,6]. The evidence suggests that worsening HF, characterized by the development of progressively escalating symptoms and signs requiring intravenous diuretic treatment in the outpatient, emergency department, or hospitalized setting, is associated with markedly worse prognosis [29,30,31,32]. The PARADIGM-HF trial showed that S/V significantly reduced both HF hospitalisation and cardiovascular mortality [9], thereby improving both prognosis and quality of life in patients with HFrEF.

These data provide a limited real-world understanding of this clinically important issue, because patients in large clinical trials are generally much more tightly monitored, clinically. However, we have recently demonstrated, in real-life data and in agreement with data from international literature, that the use of S/V in patients with HFrEF, and in maximal therapy according to guidelines, leads to an improvement in the perceived quality of life as well as increased resistance to physical effort and an improvement in cardiac performance [11,12,13,14,15,16,17,18]. In this study we compared the results obtained at 12 months with the data obtained after a further 48 months (5 years from the start of therapy with S/V). We verified that the improvements obtained after one year of therapy had not reached a plateau phase but continued to improve and were statistically significant at 5 years for LVEF and for GLS. The perceived quality of life (KCCQ) and resistance to physical effort (6MWT) continued to show improvement, but, after 5 years, it did not reach statistical significance. In addition, a further increase in patients who improved their NYHA class was obtained, thanks to the stabilization of the SBP and DBP and a further increase in the number patients of taking the maximum dose of the drug. These results mean that the benefits obtained from S/V are not limited only to the first year but continue progressively in the long term. However, the improvements that are obtained during the first year of taking the drug are clearly higher in percentage (%) (the Delta (Δ) difference in variation of each single parameter) than those obtained in the following 4 years (Figure 4).

A further finding of great importance is that no patient in our population had a worsening of HF, characterized as a reappearance of signs and symptoms requiring rehospitalization or treatment with intravenous diuretics. None of the patients required major medication changes, except an increased dose of S/V and a decreased daily dose of the diuretic, and none of the patients experienced a worsening of renal function.

### Limitations of the Study

The main limitation of the present study, due to the importance of the data presented, is that it is a single-centre, non-randomized study with a limited number of enrolled patients. Although randomization was not employed, we employed statistical methods to control for potential confounding variables and to enhance the reliability of our findings. However, our study aims to be a pilot study for much larger studies, which, if confirmed on large numbers, would change the knowledge regarding the prognosis of HFrEF.

## 5. Conclusions

This prospective observational study offers valuable 5-year follow-up data on S/V therapy in real-world HFrEF patients. While our findings show encouraging trends toward improved outcomes, we recognize that further investigation, including larger multicentre randomized trials, is necessary to safely say that S/V has brought about great changes in the prognosis of chronic HFrEF in the long term.

## Figures and Tables

**Table 1:** Patients’ characteristics at baseline.

*General Features*	*Mean ± SD*
Age, yrs	65.6 ± 10.6
Males, N (%)	60 (85.7)
Weight, kg	80.5 ± 16.1
Height, cm	168.8 ± 8
*Haemodynamic parameters*	
SBP, mmHg	125.6 ± 21.9
DBP, mmHg	75 ± 9.5
Heart Rate, beats/min	65.4 ± 12.7
*Aetiology*	
Ischaemic, N (%)	46 (65.7)
Idiopathic, N (%)	24 (34.3)
*Device*	
CRT, N (%)	10 (14.3)
ICD, N (%)	44 (62.9)
No electrical therapy, N (%)	16 (22.8)
*Co-morbidities*	
Diabetes, N (%)	34 (50)
Hypertension, N (%)	64 (94)
CKD, N (%)	6 (8.6)
Atrial Fibrillation, N (%)	18 (26)

SBP = Systolic blood pressure; DBP = Diastolic blood pressure; CRT = Cardiac resynchronization therapy; ICD = Implantable cardioverter defibrillator; CKD = Chronic kidney disease.

**Table 2:** Laboratory, clinical, and echocardiographic parameters at baseline and after 12 and 60 months of follow-up.

	Baseline	12 Months	60 Months	*p* Value
**SBP, mmHg**	125.6 (21.9)	115.6 (12.8)	119.4 (15.6)	***
**DBP, mmHg**	75 (9.5)	65.4 (7.4)	68.9 (10.8)	***
**Creatinine, mg/dL**	1.1 (0.3)	1.1 (0.3)	1.2 (0.4)	*
**Sodium, mEq/L**	139.3 (5.3)	138.6 (5.1)	139.2 (4.3)	
**Potassium, mEq/L**	4.4 (0.5)	4.4 (0.4)	4.6 (0.5)	**
**Haemoglobin, g/dL**	13.5 (1.9)	13.4 (1.9)	13.1 (2.7)	
**NTproBNP, pg/mL**	1701/1510.8	854/444	220/876	***
**BNP, pg/mL**	98/118.8	160/84.8	160/71	**
**LV EDV, mL**	188.6 (54.9)	178.1 (54.9)	171.7 (59.7)	*
**LV ESV, mL**	134.5 (41)	120.3 (41)	110.2 (45.8)	***
**LVEF, %**	29.4 (4.3)	33.4 (5.4)	37.3 (5.9)	***
**E/e’**	13 (2.9)	11.6 (2.9)	11.1 (3.4)	**
**TAPSE, mm**	20.3 (4.4)	20.9 (4.4)	21.4 (4.6)	
**Tricuspid S’, cm/s**	10.7 (3.1)	11.8 (2.7)	12.3 (2.5)	**
**GLS, %**	9.4 (1.7)	11.6 (1.8)	13.2 (1.7)	***
**6MWT, m**	256.9 (78.2)	361 (64.2)	373.8 (70.1)	***
**KCCQ**	45 (11.9)	66 (12.3)	67.2 (13)	***
**NYHA**				***
**1**	0 (0.0%)	24 (34.3%)	40 (57.1%)	
**2**	34 (48.6%)	42 (60.0%)	28 (40.0%)	
**3**	36 (51.4%)	4 (5.7%)	2 (2.9%)	

*p*-value codes: <0.001 ‘***’, 0.001 ‘**’, <0.01 ‘*’, <0.05 ‘.’, 0.1 ‘ ’ NTproBNP and BNP are expressed as median/interquartile range. SBP = Systolic blood pressure; DBP = Diastolic blood pressure; LV EDV = Left ventricular end-diastolic volume; LVEF = Left ventricular ejection fraction; TAPSE = Tricuspid annular plane excursion; GLS = Global Longitudinal Strain; 6MWT = Six Minutes Walking Test; KCCQ = Kansas City Cardiomyopathy Questionnaire; NYHA = New York Heart Association.

**Table 3:** Differences in laboratory, clinical, and echocardiographic parameters between ischaemic and idiopathic aetiology at baseline and after 12 and 60 months.

	Baseline	12 Months	60 Months	*p* Value
SBP, mmHg				# 0.478
Ischaemic Idiopathic	126.2 ± 25.6 124.5 ± 14.2	116.7 ± 14.7 112.7 ± 9.6	120.3 ± 16.9 118.1 ± 15.1	* 0.010 * 0.016
DBP, mmHg				**# 0.003**
Ischaemic Idiopathic	72.5 ± 7.9 79.5 ± 10.8	63.2 ± 7.9 68.6 ± 5.4	67 ± 10.3 70 ± 10.9	* <0.001 * <0.001
NTproBNP, pg/mL				# 0.53
Ischaemic Idiopathic	1767.5 ± 242.5 1733.1 ± 136.5	918.9 ± 178.4 867.3 ± 117.3	709.1 ± 273.1 438.2 ± 91.4	* <0.001 * <0.001
BNP				# 0.71
Ischaemic Idiopathic	132.7 ± 93.2 129.8 ± 80	159.5 ± 64.2 164.4 ± 70	203.6 ± 69.6 175.8 ± 75.9	* <0.001 * 0.002
LV EDV, mL				**# 0.036**
Ischaemic Idiopathic	183.4 ± 45.6 208.6 ± 65	170.3 ± 45.3 199.7 ± 67.6	165.1 ± 51.7 200.6 ± 72.9	* <0.001 * 0.001
LV EDVi, mL/m^2^				**# 0.024**
Ischaemic Idiopathic	96.5 ± 24.3 110.7 ± 34.6	89.5 ± 23.3 106 ± 36.3	86.2 ± 24.5 105.5 ± 36.2	* <0.001 * 0.001
LV ESV, mL				**# 0.016**
Ischaemic Idiopathic	129.7 ± 32.5 152.7 ± 50.4	114.8 ± 32.3 138.4 ± 50.6	103.8 ± 35.4 133.5 ± 56.8	* <0.001 * <0.001
LV ESVi, mL/m^2^				**# 0.010**
Ischaemic Idiopathic	68.3 ± 17.8 80.6 ± 25	60.3 ± 16.3 73.1 ± 25.6	54.2 ± 17.2 69.8 ± 26.8	* <0.001 * <0.001
LVEF, %				**# 0.017**
Ischaemic Idiopathic	29.9 ± 3.8 27.1 ± 4.8	33.9 ± 5 31 ± 5.7	38.3 ± 6.7 35.1 ± 6.6	* <0.001 * <0.001
E/e’				#0.26
Ischaemic Idiopathic	14 ± 2.4 14.6 ± 2.7	12 ± 3.2 13.6 ± 2.7	11.7 ± 3.1 12.8 ± 3.3	* <0.001 * 0.008
TAPSE, mm				# 0.98
Ischaemic Idiopathic	20.1 ± 4.7 20 ± 3.9	20.7 ± 4.8 20.3 ± 3.9	20.7 ± 5.1 21.3 ± 4.5	* <0.001 * <0.001
Tricuspid S’, cm/s				# 0.97
Ischaemic Idiopathic	10.8 ± 3.3 10.7 ± 3.3	10.8 ± 3.3 10.8 ± 3.3	10.8 ± 3.3 10.8 ± 3.3	* 0.99 * 0.015
GLS, %				# 0.53
Ischaemic Idiopathic	9.7 ± 1.8 9.9 ± 1.6	11.9 ± 2.3 11.3 ± 1.4	11.5 ± 2.7 12.7 ± 2.6	* <0.001 * <0.001
6MWT m				# 0.096
Ischaemic Idiopathic	226.9 ± 83.3 283.7 ± 85.7	352.9 ± 71.7 362 ± 50.5	364.1 ± 80.6 380.7 ± 51.7	* <0.001 * <0.001
KCCQ				# 0.12
Ischaemic Idiopathic	44.5 ± 10.4 50 ± 13.9	67.3 ± 10.5 69.6 ± 10.2	68.1 ± 10.9 72 ± 11.1	* <0.001 * <0.001
NYHA				**# 0.037**
Ischaemic Idiopathic	2.62 ± 0.5 2.3 ± 0.5	1.8 ± 0.6 1.55 ± 0.5	1.5 ± 0.6 1.36 ± 0.5	* <0.001 * <0.001

# *p* value refers to a comparison between groups over the three time-points. * *p* value refers to a comparison in the same group over the three time-points. SBP = Systolic blood pressure; DBP = Diastolic blood pressure; LV EDV = Left ventricular end-diastolic volume; LV EDVi = Left ventricular end-diastolic volume index; LV ESVi = Left ventricular end-systolic volume; LV ESVi = Left ventricular end-systolic volume index; LVEF = Left ventricular ejection fraction; TAPSE = Tricuspid annular plane excursion; GLS = Global Longitudinal Strain; 6MWT = 6 Minutes Walking Test; KCCQ = Kansas City Cardiomyopathy Questionnaire; NYHA = New York Heart Association.

## Data Availability

Data is unavailable due to privacy restrictions.
